# IgG4-related disease with eosinophil extracellular traps

**DOI:** 10.1093/qjmed/hcaf051

**Published:** 2025-02-22

**Authors:** Saori Amano, Sho Nishiguchi, Yasuhiro Mochida, Shinichi Teshima, Shigeharu Ueki

**Affiliations:** Department of General Internal Medicine, Shonan Kamakura General Hospital, Kamakura, Japan; Department of General Internal Medicine, Shonan Kamakura General Hospital, Kamakura, Japan; Department of Kidney Disease and Transplant Center, Shonan Kamakura General Hospital, Kamakura, Japan; Department of Diagnostic Pathology, Shonan Kamakura General Hospital, Kamakura, Japan; Department of General Internal Medicine and Clinical Laboratory Medicine, Akita University Graduate School of Medicine, Akita, Japan

Learning points for clinicians Significant blood and tissue eosinophilia can be observed occasionally in IgG4-related disease (IgG4-RD), which can be misdiagnosed as eosinophilic inflammatory diseases. Eosinophil extracellular trap-forming cell death and Charcot–Leyden crystals may have contributed to the refractory nature of IgG4-RD.

## Case report

**Figure 1.**
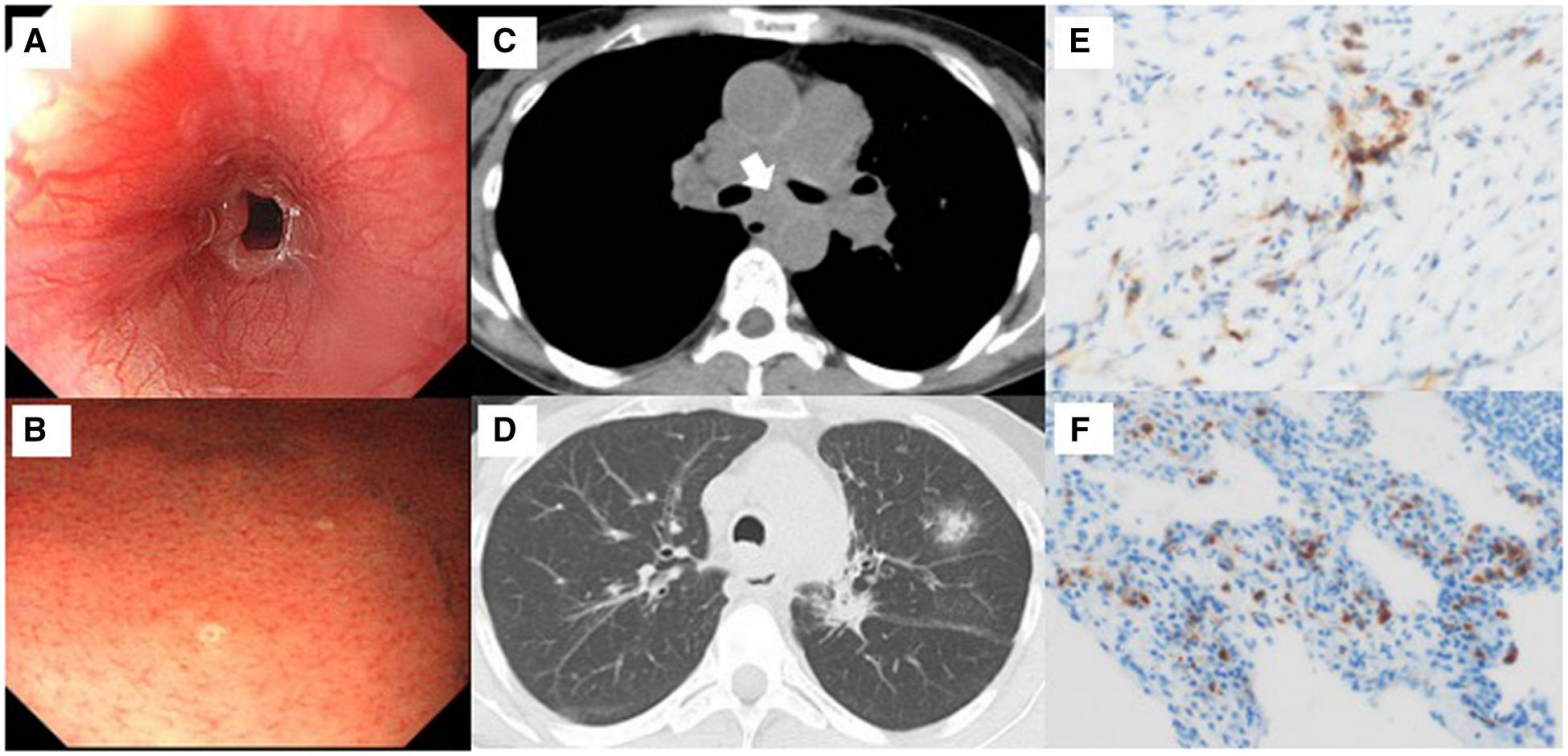
Oesophagoduodenoscopy and computed tomography (CT) images of the chest reveals (**A**) severe oesophageal stricture with normal mucous membrane, and (**B**) white deposits scattered throughout the gastric mucosa. CT image of the chest shows (**C**) mediastinal mass (arrow) that caused stenosis of the left main bronchus and oesophagus, and (**D**) new nodular shadows with a ground-glass appearance in the upper lobes. Immunohistochemical staining (**E**) of a section of the fibrosis mediastinitis revealed abundant IgG4-positive plasma cells, and (**F**) a section of the pulmonary lesion specimens obtained 10 years prior revealed many IgG4-positive plasma cells.

**Figure 2.**
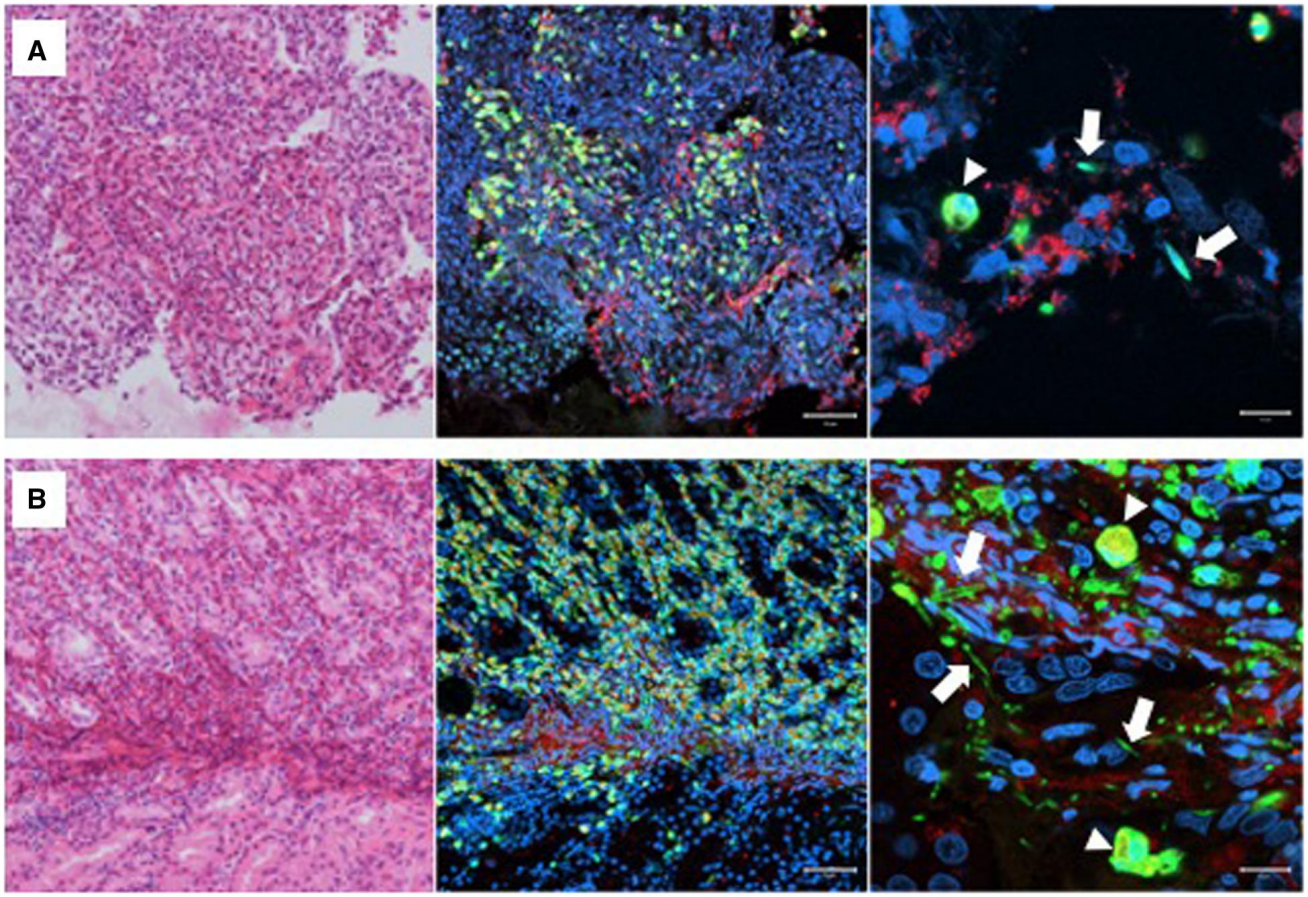
Tissue eosinophilia with cytolytic eosinophils and CLCs. Left (H&E staining) and centre images (immunofluorescence staining) are from the same section, at 200× magnification, with the scale bar representing 50 µm. The right images are magnified view at 1000×, with the scale bar indicating 10 µm. (**A**) A section of the pulmonary lesion specimens obtained 10 years prior stained for galectin-10 (green), granular protein MBP (red) and DNA (blue). Galectin-10 is clearly visualized in the cytoplasm of intact eosinophils (arrowhead) and in slender bipyramidal CLCs (arrows). Cytolytic eosinophils lose galectin-10 from their cytoplasm, with MBP granules deposited in the surrounding tissue, and their nuclei lose structure, spreading out in a net-like pattern (extracellular traps). (**B**) A section of the gastric lesion specimens. CLCs, Charcot-Leyden crystals; H&E, haematoxylin-eosin; MBP, major basic protein.

A 53-year-old woman with a history of chronic eosinophilic pneumonia, diagnosed via lung biopsy 10 years prior, together with pericarditis and asthma, presented to our hospital complaining of dysphagia. A laboratory analysis revealed mild eosinophilia (890.5/μl) and an IgG4 level of 395 mg/dl but was negative for anti-neutrophil cytoplasmic antibodies. Esophagogastroduodenoscopy revealed severe oesophageal stricture with a normal mucous membrane (Figure 1A). Scattered white deposits were also observed throughout the gastric mucosa (Figure 1B). Chest computed tomography (CT) confirmed the presence of oesophageal stenosis caused by compression from a mediastinal mass (Figure 1C). Ground-glass opacities were newly identified in the upper lobes (Figure 1D). The nodular shadows that were apparently diagnosed and treated as eosinophilic pneumonia 10 years prior were also detected in the lungs. Histological examination of the mediastinal mass revealed lymphoplasmacytic infiltration along with fibrosis but a lack of vasculitis. Immunohistochemical staining revealed 100/high power field (HPF) IgG4-positive plasma cell infiltrates; 50% of the plasma cells were positively immunolabeled with the IgG4 antibody (Figure 1E). The gastric biopsy also showed abundant IgG4-positive plasma cells. The lung biopsy performed 10 years ago was re-analysed revealing ∼80/HPF IgG4-positive plasma cells, constituting 80% of the IgG-positive plasma cells (Figure 1F). This suggested that the pulmonary disease treated as eosinophilic pneumonia for 10 years was a lesion of systemic IgG4-related disease (IgG4-RD). The gastric and lung specimens were subjected to immunofluorescence staining for eosinophil-specific proteins, galectin-10 and major basic protein (MBP). Cytolytic eosinophils with nuclear disintegration, extracellular MBP deposition, net-like nuclear DNA (extracellular traps) and galectin-10-positive Charcot–Leyden crystals (CLCs) were detected in both specimens (Figure 2). Induction therapy with 0.6 mg/kg of prednisolone was initiated, resulting in gradual improvement of dysphagia. Subsequent CT examinations revealed reduction in the mediastinal mass and improvement in the pulmonary lesions.

## Discussion

IgG4-RD is a systemic disease characterized by the formation of chronic fibroinflammatory masses or diffuse enlargement of one or more organs. Peripheral eosinophilia and eosinophilic infiltration are detected in 20–40% patients and 51–86% tissue specimens, respectively.^1^ Previous studies demonstrated the clinical relevance of blood eosinophilia in IgG4-RD. An elevated eosinophil count correlates with disease relapse and the duration until relapse occurs.^2^ Moreover, disease activity, the number of affected organs and fibrosis, may also be correlated with an increase in the eosinophil count.^3^

In recent years, eosinophil extracellular trap-forming cell death (EETosis), a type of programmed cell death, has been reported to be associated with pathological conditions of various diseases.^4^^,^^5^ Upon eosinophil activation, EETosis induces the cytolytic release of extracellular traps and intact granules, the deposition of which leads to sustained tissue damage.^5^ During EETosis, the galectin-10, which is specifically present in the cytoplasm of eosinophils, crystallizes within and outside the cells to form CLCs.^6^ Recent studies have indicated that the CLCs act as a damage-associated molecular pattern, thereby inducing further inflammation.^5^ Immunohistological studies of the lesion in the present case revealed, for the first time, not only IgG4-positive cells but also eosinophils exhibiting characteristic features of ETosis, along with the presence of CLCs. The excessive accumulation of eosinophils and their cytolytic cell death may have contributed to the refractory nature of the disease in this patient.

In conclusion, this report presents the case of a patient who was misdiagnosed as eosinophilic pneumonia 10 years ago, which ultimately was diagnosis as IgG4-RD due to the development of a mediastinal tumour. Although the patient’s tissue showed a marked sign of eosinophilic inflammation such as EETosis and CLCs, physicians should consider IgG4-RD as differential diagnosis in case of long-term, multi-organ problems associated with eosinophilia.
